# The preferences for the telemedicine and standard health care services from the perspective of the patients with schizophrenia

**DOI:** 10.1186/s12888-023-04885-8

**Published:** 2023-05-24

**Authors:** Min Li, Yanhan Chen, Xuefu Hu, Shunhong Wang

**Affiliations:** 1 College of Nursing, Chongqing Medical University, Chongqing, China; 2 Ningan mental health center, Yinchuan, Ningxia China; 3 Department of Anesthesiology, The 958th Hospital of Chinese People’s Liberation Army, Chongqing, China

**Keywords:** Schizophrenia, Telemedical services, Standard care services, Preferences

## Abstract

**Background:**

With the rapid development of telemedicine, has enabled new and various ways to deliver health care services for patients with schizophrenia. However, it is not clear that the newly emerged is better than the standard or not from the perspective of patients with schizophrenia. This study aims to explore their preferences between telemedicine and standard health care services and their associated factors.

**Methods:**

The cross-sectional study was conducted at the Ningan hospital’s inpatient department in Yinchuan, and collected socio-demographic and clinical information, the preferences regarding telemedicine (WeChat, telephone, and Email), and the standard health care services (community health center and home visit). The socio-demographic and clinical characteristics associated with the five-health care service delivery ways were assessed by descriptive analysis, and the associated impact factors of preferences of patients with schizophrenia were analyzed by multiple logistic regression.

**Results:**

Among the 300 participants, most of them chose WeChat (46.3%), some of them tended to telephone (35.4%) and community health center (11.3%), and a few of them accepted home visits (4.7%), and Email (2.3%). There are so many associated factors that affected the patients with schizophrenia to choose their favorite health care services, of which age, gender, employment, residence, and duration of illness were the independent impact factors.

**Conclusions:**

The cross-sectional study surveyed the preferences between telemedicine and standard health care services in patients with schizophrenia’s opinion, disclosed independent impact factors, as well as compared the advantage and disadvantages of these. According to our findings, the best health care services should be based on the preferences of the patients with schizophrenia and adapt to realistic conditions. This provides valuable evidence to improve the health care situation, facilitate the continuity of health care services, and achieve holistic rehabilitative outcomes for the patients with schizophrenia.

**Supplementary Information:**

The online version contains supplementary material available at 10.1186/s12888-023-04885-8.

## Introduction

Schizophrenia is regarded as being one of the most serious of all psychiatric illnesses [1], and affects about 1% of the general population around the world [2]. According to the Global Burden of Disease Study, as of 2019, there were approximately 5.5 million individuals with schizophrenia in China, ranking first in the world [3]. As early as 2011, The National Severe Mental Disorder Information Surveillance System was put into operation in our country. The official system proposes relevant policy by aggregating and analyzing the data reported by provinces and municipalities, and not provide for individuals with the health care services [4]. Almost patients with schizophrenia experience numerous relapses and ongoing impairment [5]. Obviously, patients with schizophrenia need lifelong health care services, such as reminders to take medication, sleep and diet guidance, knowledge of illness, management of psychiatric symptoms, and rehabilitation training [6]. Hence, continuous health care services are of the highest importance.

To ensure that health care services are not interrupted or hindered by external factors, the telemedicine provide more opportunities for health care services [7] and has enabled new and various ways of delivering health care services via mobile health (mHealth) systems [8], such as WeChat, QQ, Email, Facebook, What’s app, YouTube, text messaging, telephone, virtual reality (VR) systems [9–12]. Telemedicine can provide many advantages compared to the standard ways (community health center and home visit), includes accessibility, portability, as well as unconstrained by time, distance, and place [13]. Surprisingly enough, documents revealed that caregivers of patients with schizophrenia still showed a higher endorsement rate for hospital-based family intervention than WeChat-based family intervention [10]. Meanwhile, a study showed that over half of the patients with schizophrenia were not willing to participate in WeChat-based mHealth programs [14].

This has generated considerable interest in how to choose the preferred health care services for patients with schizophrenia. Neither according to the tendency of the popular or only based on the available resources of the implementation of the health care services, nor taking the perspectives of merely clinicians and caregivers of patients, which seems to be unfeasible. Patients with schizophrenia, presented with a variety of typical symptoms, including delusions, hallucinations, social withdrawal, affective blunting, and cognitive impairment [1, 15], choosing the preferred delivery way for patients with schizophrenia is the first prerequisite for long-term health care services. Some researchers have pointed out that the current health care of patients with schizophrenia, mainly from the preferences of the clinicians or the caregivers, is considerably lack of personalization and often stereotyped, not evidence-based and not targeted to the specific preference of the patients with schizophrenia [16]. So, the investigation of patients’ preferences is the first and key step for the development of mental health care systems, as well as the basis of planning mental health interventions, and is a fundamental input to form the care services [17].

However, the vast majority of studies are according to the suggestions of previous studies to implement a randomized controlled intervention trials without a survey process. It remains unclear the preferences of patients with schizophrenia regarding the telemedicine and standard health care services in china. Consequently, the main aim of our study is investigated the preferences of patients with schizophrenia with respect to the telemedicine and standard health care services, when they living in home. According to the cross-sectional data, comparing the advantages and disadvantages of the telemedicine and standard health care services and analyzing the correlate impact factors.

## Materials and methods

### Study participants

A cross-sectional study by convenient sampling was conducted at the Ningan hospital’s inpatient department in Yinchuan, Ningxia Province, China. Participants were eligible for participation if they had a primary diagnosis of the International Classification of Diseases-10 (ICD-10) for schizophrenia. They should be 18 or older, and assessed by the chief doctor be able to read, write and communicate, as well as be capable of signing informed consent. Exclusion criteria were a history of substance use disorders (besides tobacco), traumatic brain injury in the past, intellectual disabilities or any neurological disorder, and had been told by the chief doctor that he/she could not listen to the explanations about the study because of acute symptoms (e.g., hallucinations) or behavioral disorders (e.g., hurting others or selves) [18].

### Assessment tools

A survey questionnaire was developed by the authors, that based on a literature review and interviewed with psychiatrists, nurses and patients with schizophrenia [19–24]. It was to get to investigate the patients with schizophrenia preferences for health care services, when they living in home (Supplemental Data). The questionnaire comprised four sections, the first section is information about socio-demographic and clinical variables included age, gender, education, employment, residence, marital status, duration of illness, times of relapse. The second section concerned the detailed contents of health care services and consisted of 20 items in three dimensions: (a) disease management, (b) functional rehabilitation, and (c) social interaction. All items were scored by a five-point scale (1 = extremely necessary, 2 = necessary, 3 = not sure, 4 = unnecessary, 5 = extremely unnecessary). The third section in terms of the delivery way of the health care services: (a) WeChat; (b) telephone; (c) Email; (d) community health center; (e) home visit. The last section was an open comment, the detail seen in Supplementary Data. The reliability of this questionnaire is 0.921 in this study (Supplemental Data).

### Data collection

Two members of our research team, who were trained before and got the nurse practitioner license, collected data in the inpatient department of hospital. At first, we contacted the chief doctor and nurses with inclusion and exclusion criteria, they selected the eligible ones before a day. We conducted the survey with each group of 10 participants in a meeting room, after the introduction of the purpose and process of our study, all participants signed the written informed consent, subsequently completed the paper questionnaires within 15–20 min, the two members are responsible for explaining anything about the questionnaire that is in doubt or not understood. The information about duration of illness and times of relapse is mainly obtained by viewing the case file. A total of 315 questionnaires were distributed, and excluded 15 unfinished, 300 valid questionnaires were obtained, and the effective response rate was 95.2%.

### Statistical analysis

Questionnaire data were cross-checked and input into Epidata version3.1, which were analyzed by SPSS, ver.23. The categorical data used for descriptive statistics were frequencies and percentages. A Chi-square analysis was used to explore potential socio-demographic and clinical characteristics associated with the five health care services delivery ways. For better understanding the preferences of patients with schizophrenia, a multinomial logistic regression was applied to identify and compare independent impacting factors. Considering the small number of email and home visit and the statistical analysis would be affected by the value of ‘0’, those ways were categorized into ‘the other’. All tests were two-tailed, with *p* values of < 0.05 as a criterion for statistical significance.

## Results

### Socio-demographic and clinical characteristics

A total of 300 participants were enrolled in this study. Females (50.7%) were slightly more than males (49.3%). The majority were in the age of 18 ~ 30 (42.0%) and 31 ~ 44(40.0%). Education background were mainly middle school (33.0%), and high school (24.0%). Most respondents were casual employed (48.7%), married (43.0%) and lived in the urban (53.0%). Most participants had been diagnosed with schizophrenia for 3–9 years (65.7%), and experienced 3–9 times of relapse (70.0%). Details seen in Table 1.

**Table 1:** Socio-demographic and clinical characteristics of study subjects

Characteristic		Frequency(n)	Percentage(%)
Gender			
	Male	148	49.3
	Female	152	50.7
Age (in years)			
	18～30	126	42
	31～44	120	40
	≥ 45	54	18
Education			
	Primary school and below^1^	69	23
	Middle school	99	33
	High school	72	24
	Technical college	39	13
	College or higher	21	7
Employment			
	Employed	76	25.3
	Casual employed	146	48.7
	Unemployed	78	26
Marital Status			
	Single	87	29
	Married	129	43
	Divorced	84	28
Residence			
	Urban	159	53
	Rural	141	47
Duration of illness (Years)			
	≤ 2	64	21.3
	3～9	197	65.7
	≥ 10	39	13
Times of relapse			
	≤ 2	83	27.7
	3～9	209	69.7
	≥ 10	8	2.6

^1^The term “below” indicates the participants who have dropped out primary school

### The proportion of patients with schizophrenia of the five-health care services delivery ways

**Fig. 1:**
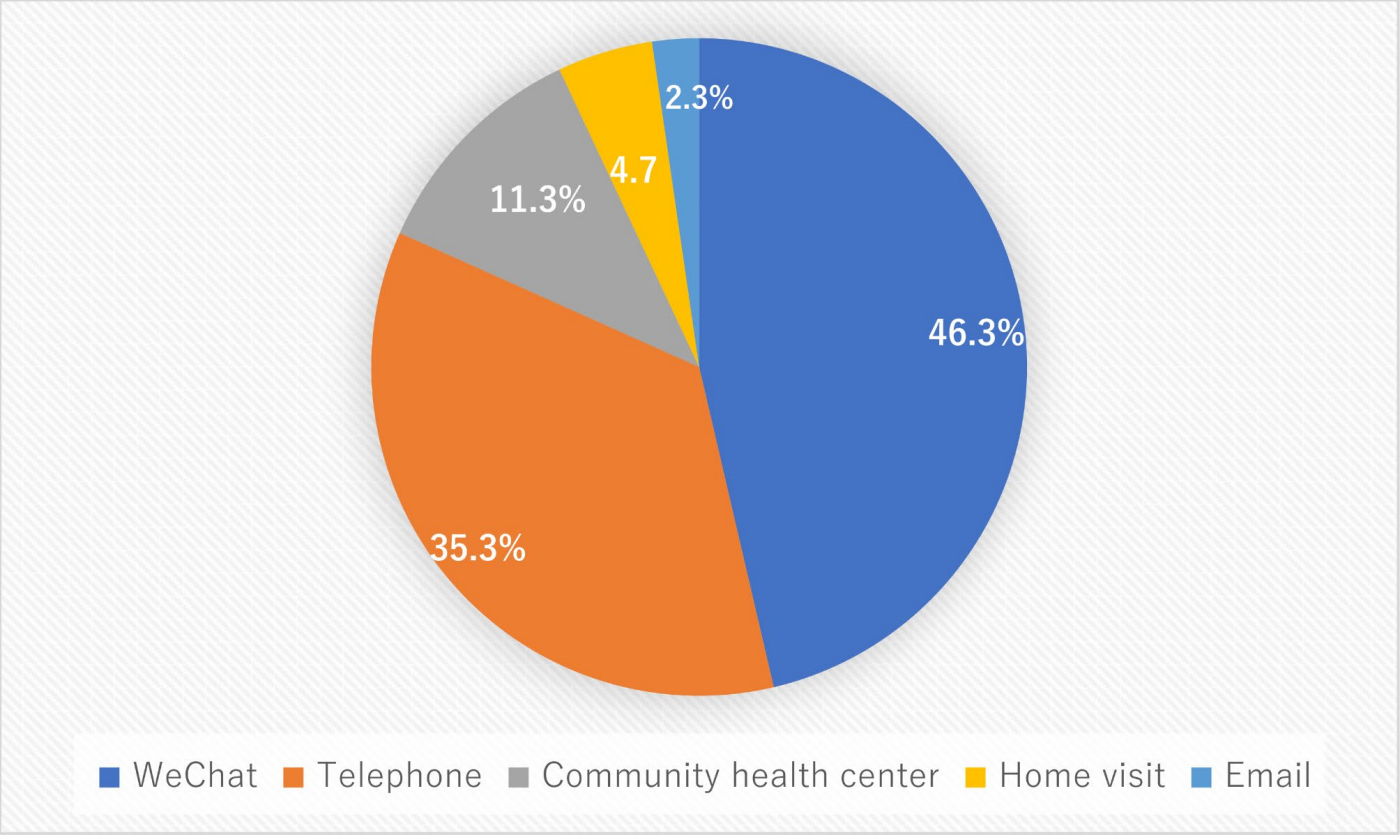
The Proportion of Patients with Schizophrenia of the Five-Health Care Services Delivery Ways

Among the 300 participants, most of them chose WeChat (46.3%), some of them tended to phone (35.4%) and community health center (11.3%), a few of them accepted home visits (4.7%) and Email (2.3%). According to the Fig. 1, the representative of the preferred ways of health care service received by the virtual or internet or in person may rely on the availability and accessibility of facility. The detailed information will be presented in the discussion section.

### Comparison of the five-health care services delivery ways in socio-demographic and clinical characteristics

Significant differences were observed in terms of age, gender, education, employment, marital status, residence, duration of illness, times of relapse, and average scores’ the second section of the questionnaire. The younger (18 ~ 30, 31 ~ 44) was more acceptable than the older (≥ 45) for WeChat and phone, while the older was more preferable the community health center and home visits (*χ2* = 49.372, *p* < 0.001). Females displayed a higher endorsement rate for WeChat than males (*χ2* = 32.392, *p* < 0.001). Higher education was accompanied by a lower acceptance for home visits (*χ2* = 39.809, *p* = 0.001). In addition, those who preferred WeChat had a higher average scores’ the second section of the questionnaire (*χ2* = 22.381, *p* = 0.002) and lived in urban (χ2 = 19.845, *p* < 0.001). The employed respondents are more likely using the WeChat than the unemployed (*χ2* = 22.027, *p* = 0.005). The single respondents showed a lowest acceptance for home visits (*χ2* = 15.323, *p* = 0.042) (Details seen in Table 2).

**Table 2:** Comparison of the five-health care services delivery ways in socio-demographic and clinical characteristics n (%)

Items	WeChat	Telephone	E-mail	Community	Home visit	*χ2*	*P*
Age (Years)						49.372	<0.001
18～30	74(53.2)	41(38.7)	2(28.6)	9(26.5)	0(0)		
31～44	56(40.3)	42(39.6)	3(42.9)	15(44.1)	4(28.6)		
≥ 45	9(6.5)	23(21.7)	2(28.5)	10(29.4)	10(71.4)		
Gender						32.392	<0.001
Male	45(32.4)	65(61.3)	6(85.7)	24(70.6)	8(57.1)		
Female	94(67.6)	41(38.7)	1(14.3)	10(29.4)	6(42.9)		
Education						39.809	0.001
Primary school and below^1^	25(18.0)	34(32.1)	0(0)	4(11.8)	6(42.8)		
Middle school	36(25.9)	41(38.7)	1(14.3)	15(44.1)	6(42.9)		
High school	38(27.3)	19(17.9)	2 (28.6)	11(32.3)	2(14.3)		
Technical college	27(19.4)	7(6.6)	3(42.9)	2(5.9)	0(0)		
College or higher	13(9.4)	5(4.7)	1 (14.3)	2 (5.9)	0(0)		
Employment						22.027	0.005
Employed	43(30.9)	19(17.9)	4(57.1)	8(23.5)	2(14.3)		
Casual employed	57(41.0)	54(50.9)	2(28.6)	21(61.8)	12(85.7)		
Unemployed	39(28.1)	33(31.2)	1(14.3)	5(14.7)	0(0)		
Marital Status						15.323	0.042
Single	41(29.5)	37(34.9)	2(28.6)	6(17.7)	1(7.1)		
Married	69(49.6)	36(34.0)	3(42.9)	14(41.2)	7(50.00)		
Divorced	29(20.9)	33(31.1)	2(28.5)	14(41.1)	6(42.9)		
Residence						19.845	<0.001
Urban	87(62.6)	41(38.7)	5(71.4)	22(64.7)	4(28.6)		
Rural	52(37.4)	65(61.3)	2 (28.6)	12(35.3)	10(71.4)		
Duration of illness (Years)						39.965	<0.001
≤ 2	40 (28.8)	14(13.2)	1 (14.3)	8(23.5)	1(7.1)		
3～9	94(67.6)	76(71.7)	5 (71.4)	16(47.1)	6(42.9)		
≥10	5(3.6)	16(15.1)	1(14.3)	10(29.4)	7(50.00)		
Times of relapse						22.381	0.002
≤ 2	47(33.8)	27(25.5)	1(14.3)	7(20.6)	1(7.1)		
3～9	92(66.2)	77(72.6)	6(85.7)	23(67.8)	11(78.6)		
≥ 10	0(0)	2(1.9)	0(0)	4(11.8)	2(14.3)		
The average score^2^						20.239	0.005
2～3	8(5.8)	17(16.1)	2(28.6)	2(5.9)	2(14.3)		
3～4	61(43.9)	56(52.8)	1(14.3)	18(52.9)	9(64.3)		
4～5	70(50.3)	33(31.1)	4(57.1)	14(41.2)	3(21.4)		

^1^The term “below” indicates the participants who have dropped out primary school

^2^The average score is average scores? the second section of the questionnaire

### Multivariate analysis to evaluate the independent impact factors to the five-health care services delivery ways

Table 3 displayed the results of the multiple logistic regression analysis for the associated factors of the five-health care services delivery ways. Age, gender, employment, residence, and duration of illness were found independent impact factors when patients with schizophrenia chose the way of health care services. The individuals with a younger age (18 ~ 30) compared to the older (≥ 45), were more acceptable for WeChat-based health care services (OR = 4.908, (1.54,15.641), *p* = 0.007), and less acceptable toward the Email or home visits-based health care services (OR = 0.133, (0.021,0.842), *p* = 0.032). Females showed a higher endorsement rate for WeChat-based health care services than males (OR = 0.299, (0.167,0.533), *p*<0.001). The participants who resided in the urban areas were more likely to use WeChat to receive health care services compared to those in the rural (OR = 2.836, (1.521,5.289), *p* = 0.001). The respondents with a shorter duration of illness (≤ 2 years) were more possibly to endorse WeChat-based health care services than those whose duration of illness was above 10 years (OR = 5.81, (1.324,25.494), *p* = 0.02). The individuals who were employed were more likely to prefer WeChat as the delivery way of health care services compared to the unemployed (OR = 2.832, (1.211,6.623), *p* = 0.016).

**Table 3:** Multivariate analysis to evaluate the independent impact factors to the five-health care services delivery ways

Variable		β	SE	Wald	*P*	OR	95%CI
WeChat	Parameter	-2.208	0.684	10.44	0.001	-	-
	Age (Years)						
	18～30	1.591	0.591	7.235	0.007	4.908	1.54～15.641
							1.54
							15.641
							1.54
							15.641
	31～44	0.949	0.565	2.817	0.093	2.582	0.853～7.817
	≥ 45(ref)	-	-	-	-	-	-
	Male	-1.209	0.296	16.666	<0.001	0.299	0.167～0.533
	Urban	1.042	0.318	10.745	0.001	2.836	1.521～5.289
	Duration of illness (Years)						
	≤ 2	1.76	0.755	5.437	0.02	5.81	1.324～25.494
	3～9	0.781	0.689	1.285	0.257	2.183	0.566～8.42
	≥ 10(ref)	-	-	-	-	-	-
	Employment						
	Employed	1.041	0.433	5.767	0.016	2.832	1.211～6.623
	Casual employed	0.515	0.364	2.002	0.157	1.674	0.82～3.417
	Unemployed(ref)	-	-	-	-	-	-
Others	Parameter	-2.399	1.22	3.867	0.049	-	-
	Age (Years)						
	18～30	-2.017	0.941	4.592	0.032	0.133	0.021～0.842
	31～44	-1.123	0.685	2.684	0.101	0.325	0.085～1.247
	≥ 45(ref)	-	-	-	-	-	-
	Male	0.306	0.536	0.325	0.568	1.358	0.475～3.883
	Urban	0.101	0.589	0.029	0.864	1.106	0.349～3.512
	Duration of illness (Years)						
	≤ 2	0.009	1.017	0.000	0.993	1.009	0.137～7.408
	3～9	-0.071	0.698	0.010	0.919	0.931	0.237～3.657
	≥ 10(ref)	-	-	-	-	-	-
	Employment						
	Employed	1.548	1.164	1.768	0.184	4.702	0.48～46.042
	Casual employed	1.779	1.106	2.588	0.108	5.922	0.678～51.708
	Unemployed(ref)	-	-	-	-	-	-
Community	Parameter	-2.619	0.847	9.556	0.002	-	-
	Age (Years)						
	18～30	0.194	0.787	0.061	0.805	1.214	0.26～5.673
	31～44	0.426	0.678	0.395	0.53	1.531	0.405～5.785
	≥ 45(ref)						
	Male	0.416	0.445	0.873	0.35	1.515	0.634～3.623
	Urban	1.415	0.469	9.117	0.003	4.115	1.643～10.309
	Duration of illness (Years)						
	≤ 2	0.115	0.815	0.02	0.888	1.122	0.227～5.546
	3～9	-1.018	0.691	2.17	0.141	0.361	0.093～1.4
	≥ 10(ref)	-	-	-	-	-	-
	Employment						
	Employed	0.617	0.704	0.768	0.381	1.853	0.466～7.363
	Casual employed	1.219	0.603	4.085	0.043	3.382	1.038～11.027
	Unemployed(ref)	-	-	-	-	-	-

* The telephone was used as the reference group

## Discussion

To the best of our knowledge, it is the first study to investigate the preferences of patients with schizophrenia regarding the telemedicine (WeChat, telephone, and Email) and the standard health care services (community health center and home visit), compare the advantages and disadvantages among those, and finally analyze the associated impact factors. A relatively high proportion of patients with schizophrenia would be interested in WeChat, while only the fewest chose Email obtaining the health care services. Telephone was acceptable, there are still many participants preferred it. For the standard ways, in terms of the community health center and home visit, very small number of patients with schizophrenia chose them. There are so many associated factors affected the patients with schizophrenia to choose their favorite delivery way of health care services, of which the age, gender, employment, residence, and duration of illness were the independent impact factors.

One major finding is that a higher percentage of individuals endorsed the WeChat-based health care services than telephone-based health care services, especially for younger, with employed, living in urban, and with the shorter course of illness. WeChat is widely used in China, with 1.13 billion monthly global active users [25]. WeChat can be used as an indispensable, typical, mature and super app of the telemedicine, including but not limited to the online consultation, patient group management and live patient education [26]. The potential benefit of it is the scalability for delivering services to more patients in China, coping the shortages in professional mental health staff [27]. The WeChat could be committed to deliver high-quality health care services, decrease loss of follow-up, improve the medication adherence, reduce disease symptoms, improve quality of life, meanwhile, relieve the financial burden (e.g. hypertension, diabetes, coronary heart disease, schizophrenia, cancer, and depression) [27–31].

However, there are also limitations and barriers to the wider usage of WeChat. The young people are usually good at the electronic device in the internet than the older; in general, it’s difficult to adapt and master how to use the internet for the latter [32]. The unemployed may be less access to pay for a smartphone with a poor economic capability. Nevertheless, broadband is an important infrastructure construction project in China in recent years, because of scattered residences and relatively low household consumption ability, by the end of 2021, 57.6% of people in rural areas were using the internet, compared to 81.3% of people in urban areas [27, 33]. That will result in the unsuitability of the WeChat-based health care services in remote village. The complex function of WeChat requires the patients with schizophrenia own a better recognitive ability, as well as a short course of illness. With development of the telemedicine, the privacy and security issues has become one of the significant concerns. The WeChat obtain so much personal information data of patients with schizophrenia, such as picture, video and text. That need be management safely and prevented a malicious disclosure of personal information [34].

That is no doubt that the rapid development of contemporary electronic device, the patients with schizophrenia have more choice for their favorite ways of health care services, The WeChat is only the one of hundreds of apps [35]. Based on the preferences of patients with schizophrenia to choose the most suitable one. Naturally, our study found that the latest is not necessarily the best for everyone.

Even though WeChat-based health care services are the most popular, telephone-based health care services still come in second place and cannot be ignored, which has a long history of implementation with rich experiences and successful cases being reported and shared by numerous previous studies [36–38]. The telephone-based health care serves are delivered by phone call and text message. The advantage compared to the WeChat is the participants do not necessarily both with smartphones and a wireless network. Thus, it is friendly to people who live in rural and remote communities. However, the disadvantages are also obvious, the communication content is conservative. So, the telephone is recommended more suitable to be combined with community health center and home visits to integrate a variety of health care services for patients with schizophrenia.

The current study observed that the individuals showed a less preferences to use Email-based health care services or home-visit-based health care services. When we conducted the investigation, many patients with schizophrenia were not familiar with Email or had not even heard of it, which may possibly attribute to the reason that Email is mainly used for formal affairs such as work and study, as well as among the users with a relatively higher education and income [39]. Alongside the widely used of WeChat in work environments, the rate of usage has a decrease tendency [40]. Although, the usage of email is wide and general in US, Hong Kong and Singapore owing to highly privacy and security [41, 42], and have demonstrated that email can increase the quality and efficiency of the health care services [43]. The Email remained underutilized in china, with an underlying value to be developed. Therefore, more research is needed to explore its development approach in the Chinese context.

Concerning the home visit, the survey concluded that only the older accepted the home visit- health care services, and few younger showed a preference for it. There are overriding reason here, the stigma of schizophrenia from the patient’s perspective, which severely affects their socialization, such as making friends, learning, and job hunting. On account of a psychiatrist comes to the patients with schizophrenia home for a follow-up visit, it means telling everyone surround that he is a maniac. The increasing evidence demonstrated that patients with schizophrenia are at high risk of suffering and internalizing stigma, which leads to self-discrimination, self-imposed isolation, and strengthening social withdrawal [44–47]. Therefore, an essential issue is to reduce the prejudice of the public towards them, that needs to educate the public widely about the concept of schizophrenia. Simultaneously, from the perspective of the psychiatric medical staff, it is difficult to carry out home-visit services, limited the severe lack of mental health care resources [48]. Apparently, the advantages of the home-visit, the patients with schizophrenia and caregivers can meet face-to-face, which facilitates accurate assessment of rehabilitation outcomes. The caregivers can get a realistic picture of the patient’s living environment and provide a practical basis for the development of rehabilitation program [49]. Thus, action is needed by official organizations such as the Centers for Disease Control and Prevention (CDC), to implement strategies that can guarantee the sufficient medical supplies and personnel, which can minimize the effect of those factors to provide the continuous health care services for patients with schizophrenia smoothly [50].

The study presented a significant difference in the attitude towards the community health center-based health care services between the patients with schizophrenia living in rural and urban areas. That may be related to there is no health care service for the remote rural population. The United Nations International Labour Organization reported that “while 56% of the global rural population lacks health coverage, only 22% of the urban population is not covered,” further compounded by rural health work-force shortages resulting in a lack of access to urgently needed care for half the global rural population [51]. The most advantages of the community health center are alleviating the financial burden, some regular medicines are free of charge [4]. However, the national severe mental disorder information surveillance system revealed that the patients with severe mental disorders did not receive professional therapy or were not treated very well in the community health center [52]. The main reasons are summarized as follows: (a) The inherent complexity and intractability of schizophrenia, as well as the characteristic psychotic symptoms, are extremely different from usual chronic disease such as hypertension and diabetes; (b) In addition to conventional antipsychotic medication, professional psychosocial, cognitive, and behavioral interventions or therapy, as well as social-vocational training are in demand. Those call for numerous personnel, material, and financial resources; (c) Stigma and discrimination coming from the public and even health professionals prevent patients when the patients with schizophrenia seek professional medical assistant [53]. Although, health professionals are medically trained, they are prone to hold negative attitudes and label patients with schizophrenia [54]. Analyzed the current state of the community health center has turned our perceptions upside down. Despite the long development and abundant experience of it, there are still many dilemmas and unsatisfactory results. Although, with so many difficulties, we still should be committed to all patients with schizophrenia access to the health care services. So, when we revealed the strengths and weaknesses of each way, followed the preferences of the patients with schizophrenia. We can combine a variety of ways to deliver the health care services and alleviate the gap among these.

## Limitations

A few limitations in this study must be noted. First, the cross-sectional design cannot support causal inferences or temporal ordering between the predictor variables of preferences and correlates. So future longitudinal study is needed. The study sample was sourced from one hospital may have led to some biases in the data and should be expanded for future study. The self-designed questionnaire lack of standardized and relevant assessment. The study without detailed data on psychopathology to better understand patients with schizophrenia choices, should be considerate in the future.

## Conclusions

The cross-sectional study surveyed the preferences between the telemedicine and standard health care services in patients with schizophrenia’s opinion, disclosed independent impact factors, as well as compared the advantage and disadvantage of these. According to our findings, the best health care services should be based on the preferences of the patients with schizophrenia and adapt to realistic conditions. Which provides valuable evidences to improve the health care situation, facilitate continuity of health care services, and achieve holistic rehabilitative outcomes for the patients with schizophrenia.

## Electronic supplementary material

Below is the link to the electronic supplementary material.

Supplementary Material 1

Supplementary Material 2

## Data Availability

The datasets used and/or analyzed during the present study are available from the corresponding author on reasonable request.

## References

[1] Jauhar S, Johnstone M, McKenna PJ, Schizophrenia. Lancet. 2022 Jan 29;399 (10323): 473–486. doi: 10.1016/S0140-6736(21)01730-X. PMID: 35093231. 10.1016/S0140-6736(21)01730-X 35093231

[2] Ferrarelli F. Sleep Abnormalities in Schizophrenia: State of the Art and Next Steps. Am J Psychiatry. 2021 Oct 1;178(10):903–913. doi: 10.1176/appi.ajp. 2020.20 070968. Epub 2021 Mar 17. PMID: 33726524; PMCID: PMC8446088. 10.1176/appi.ajp.2020.20070968 PMC8446088 33726524

[3] IHME. Global Health Data Exchange GBD 2019. (2019). Available online at: https://vizhub.healthdata.org/gbd-compare/ (accessed August 30, 2021). [Google Scholar].

[4] Peng X, Wang S, Bi J, You L, Zhou Z, Tan W, Xie H, Hu C, Ng CH, Liu T. Gender differences in socio-demographics, clinical characteristic and quality of life in patients with schizophrenia: a community-based study in Shenzhen. Asia Pac Psychiatry 2021 Jun;13(2):e12446. doi: 10.1111/appy.12446. Epub 2020 Dec 16. PMID: 33327044. 10.1111/appy.12446 33327044

[5] Correll CU, Howes OD, Treatment-Resistant Schizophrenia. Definition, Predictors, and Therapy Options. J Clin Psychiatry. 2021 Sep 7;82(5):MY20096AH1C. doi: 10.4088/JCP.MY20096AH1C. PMID: 34496461. 10.4088/JCP.MY20096AH1C 34496461

[6] Martinelli A, Ruggeri M (2020). The impact on psychiatric rehabilitation of personal recovery-oriented approach. J Psychopathol.

[7] Cao J, Lim Y, Sengoku S, Guo X, Kodama K. Exploring the Shift in International Trends in Mobile Health Research From 2000 to 2020: Bibliometric Analysis. JMIR Mhealth Uhealth. 2021 Sep 8;9(9):e31097. doi: 10.2196/31097. PMID: 34494968; PMCID: PMC8459219. 10.2196/31097 PMC8459219 34494968

[8] Noorbergen TJ, Adam MTP, Teubner T, Collins CE. Using co-design in Mobile Health System Development: a qualitative study with experts in co-design and Mobile Health System Development. JMIR Mhealth Uhealth 2021 Nov 10;9(11):e27896. doi: 10.2196/27896. PMID: 34757323; PMCID: PMC8663505. 10.2196/27896 PMC8663505 34757323

[9] Wang Y, Min J, Khuri J, Xue H, Xie B, A Kaminsky L, Cheskin J. L. Effectiveness of Mobile Health Interventions on Diabetes and Obesity Treatment and Management: Systematic Review of Systematic Reviews. JMIR Mhealth Uhealth. 2020 Apr 28;8(4):e15400. doi: 10.2196/154 00. PMID: 32343253; PMCID: PMC7218595. 10.2196/15400 PMC7218595 32343253

[10] Zhang X, Liu B, Zang D, Li Y, Xiao S, Yu Y. Preferences for WeChat-Based and hospital-based family intervention among caregivers of people living with Schizophrenia. Patient Prefer Adherence 2022 Mar 5;16:635–45. doi: 10.2147/PPA.S338936. PMID: 35283626; PMCID: PMC8906870. 10.2147/PPA.S338936 PMC8906870 35283626

[11] Xu DR, Xiao S, He H, Caine ED, Gloyd S, Simoni J, Hughes JP, Nie J, Lin M, He W, Yuan Y, Gong W. Lay health supporters aided by mobile text messaging to improve adherence, symptoms, and functioning among people with schizophrenia in a resource-poor community in rural China (LEAN): A randomized controlled trial. PLoS Med. 2019 Apr 23;16(4):e1002785. doi: 10.1371/journal.pmed.1002785. PMID: 31013275; PMCID: PMC6478272. 10.1371/journal.pmed.1002785 PMC6478272 31013275

[12] Freeman D, Lambe S, Kabir T, Petit A, Rosebrock L, Yu LM, Dudley R, Chapman K, Morrison A, O’Regan E, Aynsworth C, Jones J, Murphy E, Powling R, Galal U, Grabey J, Rovira A, Martin J, Hollis C, Clark DM, Waite F. ; gameChange Trial Group. Automated virtual reality therapy to treat agoraphobic avoidance and distress in patients with psychosis (gameChange): a multicentre, parallel-group, single-blind, randomised, controlled trial in England with mediation and moderation analyses. Lancet Psychiatry 2022 May;9(5):375–88. doi: 10.1016/S2215-0366(22)00060-8. Epub 2022 Apr 5. PMID: 35395204; PMCID: PMC9010306. 10.1016/S2215-0366(22)00060-8 PMC9010306 35395204

[13] Buck B, Nguyen J, Porter S, Ben-Zeev D, Reger GM. FOCUS mHealth Intervention for Veterans With Serious Mental Illness in an Outpatient Department of Veterans Affairs Setting: Feasibility, Acceptability, and Usability Study. JMIR Ment Health. 2022 Jan 28;9(1):e26049. doi: 10.2196/26049. PMID: 35089151; PMCID: PMC8838564. 10.2196/26049 PMC8838564 35089151

[14] Xiao S, Li T, Zhou W, Shen M, Yu Y. WeChat-based mHealth intention and preferences among people living with schizophrenia. PeerJ 2020 Dec 16;8:e10550. doi: 10.7717/peerj.10550. PMID: 33362979; PMCID: PMC7749651. 10.7717/peerj.10550 PMC7749651 33362979

[15] Citrome L, Belcher E, Stacy S, Suett M, Mychaskiw M, Salinas GD. Perceived Burdens and Educational needs of caregivers of people with Schizophrenia: results of a National Survey Study. Patient Prefer Adherence 2022 Jan 21;16:159–68. doi: 10.2147/PPA.S326290. PMID: 35087268; PMCID: PMC8789274. 10.2147/PPA.S326290 PMC8789274 35087268

[16] Tortorella A. We should improve personalization of management in patients with a diagnosis of Schizophrenia. J Clin Med. 2021 Dec;30(1):184. 10.3390/jcm11010184. PMID: 35011925; PMCID: PMC8745754. 10.3390/jcm11010184 PMC8745754 35011925

[17] Stefanatou P, Tsompanaki E, Lavdas M, Giannouli E, Ralli I, Kalogerakou S, Anyfandi E, Stylianidis S, Stefanis N, Mavreas V, Konstantakopoulos G. Patient-reported needs predict perceived psychosocial disability and quality of life beyond symptom severity in schizophrenia. Disabil Rehabil. 2022 Feb 22:1–9. doi: 10.1080/09638288.2022.2040610. Epub ahead of print. PMID: 35191793. 10.1080/09638288.2022.2040610 35191793

[18] Lee K, Bejerano IL, Han M, Choi HS. Willingness to use smartphone apps for lifestyle management among patients with schizophrenia. Arch Psychiatr Nurs. 2019 Aug;33(4):329–36. 10.1016/j.apnu.2019.01.002. Epub 2019 Feb 8. PMID: 31280776. 10.1016/j.apnu.2019.01.002 31280776

[19] Lin LE, Lo SC, Liu CY, Chen SC, Wu WC, Liu WI. Effectiveness of needs-oriented hospital discharge planning for caregivers of patients with Schizophrenia. Arch Psychiatr Nurs. 2018 Apr;32(2):180–7. 10.1016/j.apnu.2017. 10.013. Epub 2017 Oct 16. PMID: 29579510. 10.1016/j.apnu.2017.10.013 29579510

[20] Tsui MCM, Tsang HWH. Views of people with schizophrenia and their caregivers towards the needs for psychiatric rehabil-itation in urban and rural areas of mainland China. Psychiatry Res. 2017 Dec;258:72–7. 10.1016/j.psychres.2017.09.052. Epub 2017 Sep 25. PMID: 28988122. 10.1016/j.psychres.2017.09.052 28988122

[21] Wazni L, Gifford W. Addressing Physical Health Needs of Individuals With Schizophrenia Using Orem’s Theory. J Holist Nurs. 2017 Sep;35(3):271–279. doi: 10.1177/0898010116658366. Epub 2016 Jul 11. PMID: 27406850. 10.1177/0898010116658366 27406850

[22] Eskelinen S, Sailas E, Joutsenniemi K, Holi M, Koskela TH, Suvisaari J. Multiple physical healthcare needs among outpatients with schizophrenia: findings from a health examination study. Nord J Psychiatry. 2017 Aug;71(6):448–54. Epub 2017 May 12. PMID: 28497707. 10.1080/08039488.2017.1319497 28497707

[23] Ben-Zeev D, Scherer EA, Gottlieb JD, Rotondi AJ, Brunette MF, Achtyes ED, Mueser KT, Gingerich S, Brenner CJ, Begale M, Mohr DC, Schooler N, Marcy P, Robinson DG, Kane JM. mHealth for Schizophrenia: Patient Engagement With a Mobile Phone Intervention Following Hospital Discharge. JMIR Ment Health. 2016 Jul 27;3(3): e34. doi: 10.2196/mental.6348. PMID: 27465803; PMCID: PMC 4999306. 10.2196/mental.6348 PMC4999306 27465803

[24] Terp M, Jørgensen R, Laursen BS, Mainz J, Bjørnes CD. A Smartphone App to Foster Power in the Everyday Management of Living with Schizophrenia: Qualitative Analysis of Young Adults’ Perspectives. JMIR Ment Health. 2018 Oct 1;5(4):e10157. doi: 10.2196/10157. PMID: 30274966; PMCID: PMC6231723. 10.2196/10157 PMC6231723 30274966

[25] Tian Y, Zhang S, Huang F, Ma L. Comparing the Efficacies of Telemedicine and Standard Prenatal Care on Blood Glucose Control in Women With Gestational Diabetes Mellitus: Randomized Controlled Trial. JMIR Mhealth Uhealth. 2021 May 25;9(5): e22881. doi: 10.2196/22881. PMID: 33783365; PMCID: PMC8188321. 10.2196/22881 PMC8188321 33783365

[26] Luo T, Li M, Williams D, Fritz J, Phillippi S, Yu Q, Kantrow S, Chen L, Chen Y, Beiter K, Tseng TS. Urban and Rural Disparities in a WeChat-Based Smoking Cessation Intervention among Chinese Smokers. Int J Environ Res Public Health. 2021 Jun 23;18(13):6731. doi: 10.3390/ijerph18136731. PMID: 34201450; PMCID: PMC8268404. 10.3390/ijerph18136731 PMC8268404 34201450

[27] Huang L, Li VW, Yang T, Liu J, Murphy J, Michalak EE, Wang Z, Ng C, Yatham L, Chen J, Lam RW. Mobile Health Applications for Depression in China: a systematic review. Cureus 2022 Jul 26;14(7):e27299. doi: 10.7759/cureus.27299. PMID: 35903483; PMCID: PMC9320610. 10.7759/cureus.27299 PMC9320610 35903483

[28] Gao XT, Huang KC, Cui X, Zhou CM. WeChat-assisted health education improves care ability, reduces care burden and improves quality of life of parents of infants after enterostomy. J Paediatr Child Health. 2021 Jul;57(7):1067–1071. doi: 10.1111/jpc.15399. Epub 2021 Mar 5. PMID: 33667028. 10.1111/jpc.15399 33667028

[29] Chen X, Zhou X, Li H, Li J, Jiang H. The value of WeChat application in chronic diseases management in China. Comput Methods Programs Biomed. 2020 Nov;196:105710. 10.1016/j.cmpb.2020.105710. Epub 2020 Aug 14. PMID: 32858284. 10.1016/j.cmpb.2020.105710 32858284

[30] Mao J, Xie L, Zhao Q, Xiao M, Tu S, Sun W, Zhou T. Demand analysis of an intelligent medication administration system for older adults with chronic diseases based on the Kano model. Int J Nurs Sci. 2021 Dec 24;9(1):63–70. doi: 10.1016/j.ijnss.2021.12.012. PMID: 35079606; PMCID: PMC8766777. 10.1016/j.ijnss.2021.12.012 PMC8766777 35079606

[31] Zhu X, Li M, Liu P, Chang R, Wang Q, Liu J. A mobile health application-based strategy for enhancing adherence to antipsychotic medication in schizophrenia. Arch Psychiatr Nurs. 2020 Dec;34(6):472–80. Epub 2020 Aug 5. PMID: 33280669. 10.1016/j.apnu.2020.08.001 33280669

[32] Yu-Ting Y, Miao Y, Yong-Wei Y, Qiong Y, Ting L. Exploring urban empty-nesters’ using WeChat influencing factors and quality of life: a qualitative descriptive study. Geriatr Nurs 2022 Nov-Dec;48:183–9. doi: 10.1016/j.gerinurse.2022.10.008. Epub 2022 Oct 20. PMID: 36272341. 10.1016/j.gerinurse.2022.10.008 36272341

[33] Liu Y, Shen T, Nagai Y, Wu W. Can the income level of rural residents be improved by the chinese “Broadband Village?“: evidence from a regression discontinuity design of the six pilot provinces. PLoS One 2021 Apr 20;16(4):e0248079. doi: 10.1371/journal.pone.0248079. PMID: 33878106; PMCID: PMC8057606. 10.1371/journal.pone.0248079 PMC8057606 33878106

[34] Aljedaani B, Babar MA. Challenges With Developing Secure Mobile Health Applications: Systematic Review. JMIR Mhealth Uhealth. 2021 Jun 21;9(6):e15654. doi: 10.2196/15654. PMID: 34152277; PMCID: PMC8277314. 10.2196/15654 PMC8277314 34152277

[35] Tan Y, Teng Z, Qiu Y, Tang H, Xiang H, Chen J (2020). Potential of mobile technology to relieve the urgent mental health needs in China: web-based survey. JMIR Mhealth Uhealth.

[36] van den Berg N, Grabe HJ, Baumeister SE, Freyberger HJ, Hoffmann W (2015). A telephone- and text message-based Telemedicine Concept for patients with Mental Health Disorders: results of a Randomized Controlled Trial. Psychother Psychosom.

[37] Schulze LN, Stentzel U, Leipert J, Schulte J, Langosch J, Freyberger HJ, Hoffmann W, Grabe HJ, van den Berg N. Improving Medication Adherence With Telemedicine for Adults With Severe Mental Illness. Psychiatr Serv. 2019 Mar 1;70(3):225–228. doi: 10.1176/appi.ps.201800286. Epub 2019 Jan 17. PMID: 30651059. 10.1176/appi.ps.201800286 30651059

[38] Stentzel U, van den Berg N, Moon K, Schulze LN, Schulte J, Langosch JM, Hoffmann W, Grabe HJ. Telemedical care and quality of life in patients with schizophrenia and bipolar disorder: results of a randomized controlled trial. BMC Psychiatry 2021 Jun 29;21(1):318. doi: 10.1186/s12888-021-03318-8. PMID: 34187420; PMCID: PMC8243575. 10.1186/s12888-021-03318-8 PMC8243575 34187420

[39] Zhu J, Bruhn A, Yuan C, Wang L. Comparing the effects of videoconference and email feedback on treatment integrity. J Appl Behav Anal. 2021 Apr;54(2):618–635. doi: 10.1002/jaba.810. Epub 2021 Jan 20. PMID: 33472275. 10.1002/jaba.810 33472275

[40] Firms, We Chat Statistics. 2020. Available online: https://99firms.com/blog/wechat-statistics/#gref (accessed on 18 May 2020).

[41] Kindratt TB, Allicock M, Atem F, Dallo FJ, Balasubramanian BA. Email Patient-Provider Communication and Cancer Screenings Among US Adults: Cross-sectional Study. JMIR Cancer. 2021 Jul 30;7(3):e23790. doi: 10.2196/23790. PMID: 34328421; PMCID: PMC8367146. 10.2196/23790 PMC8367146 34328421

[42] Zheng S, Chan SKW, Lee J. Managing treatment resistance in schizophrenia: a joint study in Hong Kong and Singapore. Front Psychiatry 2022 Oct 20;13:1005373. doi: 10.3389/fpsyt.2022.1005373. PMID: 36339860; PMCID: PMC9631784. 10.3389/fpsyt.2022.1005373 PMC9631784 36339860

[43] Antoun J. Electronic mail communication between physicians and patients: a review of challenges and opportunities. Fam Pract. 2016 Apr;33(2):121–6. 10.1093/fampra/cmv101. [PubMed] [CrossRef] [Google Scholar]. 10.1093/fampra/cmv101 26711957

[44] Luo H, Li Y, Yang BX, Chen J, Zhao P. Psychological interventions for personal stigma of patients with schizophrenia: a systematic review and network meta-analysis. J Psychiatr Res 2022 Apr;148:348–56. doi:10.1016/j.jpsychires.2022.02.010. Epub 2022 Feb 16. PMID: 35202995. 10.1016/j.jpsychires.2022.02.010 35202995

[45] Barlati S, Morena D, Nibbio G, Cacciani P, Corsini P, Mosca A, Deste G, Accardo V, Turrina C, Valsecchi P, Vita A. Internal-ized stigma among people with schizophrenia: Relationship with socio-demographic, clinical and medication-related fea-tures. Schizophr Res. 2021 Jun 25:S0920-9964(21)00219-X. doi: 10.1016/j.schres.2021.06.007. Epub ahead of print. PMID: 34183209. 10.1016/j.schres.2021.06.007 34183209

[46] Komatsu H, Ono T, Onoguchi G, Tomita H, Kakuto Y. Mediating effects of self-stigma and depression on the association be-tween autistic symptoms and recovery in patients with schizophrenia-spectrum disorders: a cross-sectional study. BMC Psychiatry. 2021 Sep 23;21(1):464. doi: 10.1186/s12888-021-03472-z. PMID: 34556056; PMCID: PMC8461904. 10.1186/s12888-021-03472-z PMC8461904 34556056

[47] Dhungana S, Tulachan P, Chapagai M, Pant SB, Lama PY, Upadhyaya S. Internalized stigma in patients with schizophrenia: A hospital-based cross-sectional study from Nepal. PLoS One. 2022 Mar 11;17(3):e0264466. doi: 10.1371/journal.pone.0264466. PMID: 35275907; PMCID: PMC8916637. 10.1371/journal.pone.0264466 PMC8916637 35275907

[48] Thornicroft G, Semrau M. Health system strengthening for mental health in low- and middle-income countries: introduction to the Emerald programme. BJPsych Open. 2019 Aug 6;5(5):e66. doi: 10.1192/bjo.2019.9. PMID: 31685066; PMCID: PMC6688463. 10.1192/bjo.2019.9 PMC6688463 31685066

[49] Mashimo I, Yotsumoto K, Fujimoto H, Hashimoto T. Effects of Home-visit Occupational Therapy using a Management Tool for Daily Life performance on severe Mental illness: a Multicenter Randomized Controlled Trial. Kobe J Med Sci 2020 Dec 15;66(4):E119–28. PMID: 33994515; PMCID: PMC8212806. PMC8212806 33994515

[50] GBD 2019 Mental Disorders Collaborators. Global, regional, and national burden of 12 mental disorders in 204 countries and territories, 1990–2019: a. Lancet Psychiatry. 2022 Feb;9(2):137–50. 10.1016/S2215-0366(21)00395-3. Epub 2022 Jan 10. PMID: 35026139; PMCID: PMC8776563. systematic analysis for the Global Burden of Disease Study 2019. 10.1016/S2215-0366(21)00395-3 PMC8776563 35026139

[51] Stockton DA, Fowler C, Debono D, Travaglia J. World Health Organization building blocks in rural community health ser-vices: An integrative review. Health Sci Rep. 2021 Mar 9;4(2):e254. doi: 10.1002/hsr2.254. PMID: 33732894; PMCID: PMC7942400. 10.1002/hsr2.254 PMC7942400 33732894

[52] Wang D, Ma J, Tan L, Chen Y, Li X, Tian X, Zhou X, Liu X (2017). Epidemiology of severe mental illness in Hunan province in Cen-tral China during 2014–2015: a multistage cross-sectional study. PLoS ONE.

[53] Chen L, Zhao Y, Tang J, Jin G, Liu Y, Zhao X, Chen C, Lu X. The burden, support and needs of primary family caregivers of people experiencing schizophrenia in Beijing communities: a qualitative study. BMC Psychiatry 2019 Feb 20;19(1):75. doi: 10.1186/s12888-019-2052-4. PMID: 30786852; PMCID: PMC6381607. 10.1186/s12888-019-2052-4 PMC6381607 30786852

[54] Lin CH, Lai TY, Chen YJ, Lin SK. Social distance towards schizophrenia in health professionals. Asia Pac Psychiatry. 2021 Dec 16:e12506.doi:10.1111/appy.12506. Epub ahead of print. PMID: 34915596. 10.1111/appy.12506 34915596

