# Supplementary figures and images for: The preferences for the telemedicine and standard health care services from the perspective of the patients with schizophrenia
Source: BMC Psychiatry. 2023 May 24;23:361. doi: 10.1186/s12888-023-04885-8 (PMC10210282; doi:10.1186/s12888-023-04885-8)

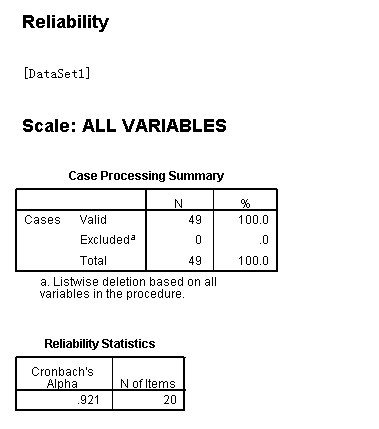

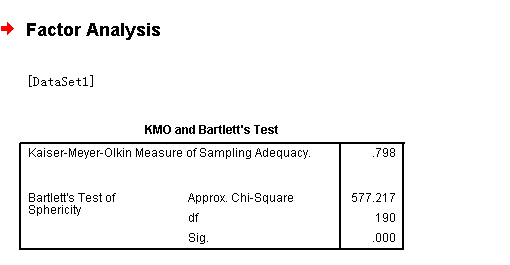

Supplement: Supplementary file 1 — Supplementary Material 1 [file 12888_2023_4885_MOESM1_ESM.docx]
