# Supplementary material for: The preferences for the telemedicine and standard health care services from the perspective of the patients with schizophrenia
Source: BMC Psychiatry. 2023 May 24;23:361. doi: 10.1186/s12888-023-04885-8 (PMC10210282; doi:10.1186/s12888-023-04885-8)
Supplement: Supplementary file 2 — Supplementary Material 2 [file 12888_2023_4885_MOESM2_ESM.doc]

Questionnaire on preferences of the health care services of patients with schizophrenia

Dear participants,

This questionnaire is to understand your preferences of the health care services in the process of stable maintenance treatment out of hospital. Please read each item carefully and tick the number of options according to your view. Any option is acceptable and all the information in this survey will be kept confidential. Thank you for your cooperation!

Age: ______ Gender: ______ Education: ______ Employment: ______

Residence: urban / rural Marital status: single / married / divorced

Duration of illness (Years): Times of relapse:

| **Item** | Extremely necessary | Necessary | Not sure | Unnecessary | Extremely unnecessary |
| --- | --- | --- | --- | --- | --- |
| 1. Knowing how to take the drugs after discharge | 5 | 4 | 3 | 2 | 1 |
| 2. Knowing how to take different drugs | 5 | 4 | 3 | 2 | 1 |
| 3. Knowing the side effects of each drug | 5 | 4 | 3 | 2 | 1 |
| 4. Knowing how to deal with side effects of drugs | 5 | 4 | 3 | 2 | 1 |
| 5. Knowing the harm of drug withdrawal at will | 5 | 4 | 3 | 2 | 1 |
| 6. Knowing the precautions of diet during taking drugs | 5 | 4 | 3 | 2 | 1 |
| 7. Reminding you to take drugs on time | 5 | 4 | 3 | 2 | 1 |
| 8. Reminding you of regular follow-up visits | 5 | 4 | 3 | 2 | 1 |
| 9. Knowing how to recognize the early signs of disease recurrence | 5 | 4 | 3 | 2 | 1 |
| 10. Your family members to identify the changes and recurrence of your disease | 5 | 4 | 3 | 2 | 1 |
| 11. Designing a rest schedule for you | 5 | 4 | 3 | 2 | 1 |
| 12. Knowing how to deal with negative emotions | 5 | 4 | 3 | 2 | 1 |
| 13.Cognitive rehabilitation (such as memory enhancement, attention maintenance, language fluency, word fluency, etc.) | 5 | 4 | 3 | 2 | 1 |
| 14.Social rehabilitation (such as vocational training and housework)? | 5 | 4 | 3 | 2 | 1 |
| 15. Knowing the methods of sports activities that are helpful for your recovery? | 5 | 4 | 3 | 2 | 1 |
| 16. Knowing how to improve your social communication skills? | 5 | 4 | 3 | 2 | 1 |
| 17. Knowing the methods and ways to obtain social support? | 5 | 4 | 3 | 2 | 1 |
| 18. Joining our client club? | 5 | 4 | 3 | 2 | 1 |
| 19. Keeping contact with medical personnel through WeChat? | 5 | 4 | 3 | 2 | 1 |
| 20. Keeping contact with other clients through WeChat? | 5 | 4 | 3 | 2 | 1 |

21. Which way do you most expect to deliver the health care services (single choice)?

1. WeChat B. telephone C. Email D. community health center E. home visit

22. What else health care services do you want after dismissed from hospital? (not included in over-mentioned questions)?

__________________________________________________________________________________________________________________________________________________________________
